# Rose hip supplementation increases energy expenditure and induces browning of white adipose tissue

**DOI:** 10.1186/s12986-016-0151-5

**Published:** 2016-12-06

**Authors:** Michele Cavalera, Ulrika Axling, Karin Berger, Cecilia Holm

**Affiliations:** Department of Experimental Medical Science, Lund University, Lund, Sweden

**Keywords:** Obesity, Diet, Browning, Energy expenditure, Rose hip

## Abstract

**Background:**

Overweight and obesity are widespread chronic disorders defined as excessive fat accumulation, and are major risk factors for several chronic diseases including type 2 diabetes, coronary heart disease, high blood pressure and fatty liver. Changes in lifestyle such as increased physical activity and a healthy diet can be crucial tools for treating obesity. Intake of rose hip, the fruit of several plants belonging to the *Rosaceae* family, has been shown to reduce body fat mass and prevent body weight gain. Thus, the aim of the study was to elucidate potential mechanisms through which rose hip inhibit diet-induced obesity.

**Methods:**

C57BL/6 J mice were fed a high fat diet with (RH) or without (CTR) rose hip supplementation for three months. In vivo indirect calorimetry was monitored, as well as gene expression and protein levels of different adipose depots.

**Results:**

Although no differences in energy intake were found compared to the CTR group, RH prevented body weight gain and lowered blood glucose, insulin and cholesterol levels. Indirect calorimetry showed that RH-fed mice have significantly higher EE during the dark phase, despite comparable voluntary activity. Moreover, when challenged with treadmill running, RH-fed mice exhibited higher metabolic rate. Therefore, we hypothesized that RH could stimulate the brown adipose tissue (BAT) thermogenic capacity or may induce browning of the white adipose tissue (WAT). Compared to the CTR group, gene expression and protein levels of some brown and “brite” markers, together with genes able to promote brown adipocyte differentiation and thermogenesis (such as *ucp1, tbx15*, *bmp7,* and *cidea*), as well as phosphorylation of AMPK, was increased in WAT (but not in BAT) of RH-fed mice.

**Conclusions:**

Taken together these results indicate that dietary rose hip prevents body weight gain by increasing whole body EE and inducing browning of WAT. Thus, it has potential therapeutic implication for treatment of obesity and related metabolic disorders.

## Background

Obesity is a condition that leads to premature death due to several comorbidities, such as cardiovascular disease [1], type 2 diabetes [2], hypertension [3] and cancer [4, 5]. Adipose tissue consists of two functionally and morphologically distinct tissue types, the WAT and BAT [6]. One of the main roles of the unilocular WAT is the storage of triglycerides and release of fatty acids and adipocytokines, while the multilocular BAT also dissipates energy in the form of heat through the constitutively expressed UCP1 [7, 8]. Another sub-type of adipocytes with thermogenic capacity can appear within the WAT in response to different stimuli [9]. These cells have been named “brite” (brown in white), “beige”, “inducible” or “recruitable”, and attain features typical of the BAT such as high mitochondrial content, multilocular lipid droplets and the ability to express BAT-specific genes [9–12]. Since the discovery of BAT also being present in adult humans [13–17], increasing its thermogenic capacity or inducing the conversion of white into beige fat could be a therapeutic strategy for obesity, diabetes and metabolic disorders [8].

Many factors like drugs, cold exposure, adrenergic stimulation and exercise are able to induce browning of the white fat [8, 11, 18]. Some dietary components can also affect energy homeostasis. For instance resveratrol [19], curcumin [20] or conjugated linoleic acids [21] have been shown to reduce adiposity and induce brown-like adipocyte in WAT.

Rose hip (RH) is the fruit of several plants of the genus *Rosa*, belonging to the *Rosaceae* family, and it is rich in ascorbic acid [22], phenolic compounds [23] and carotenoids [24]. It exerts anti-obesity, anti-inflammatory and anti-oxidative effects both in vivo and in vitro [25–30], and has beneficial and pain-relieving properties in subjects with arthritis [31–34]. Obesity results from an imbalance between energy intake (EI) and energy expenditure (EE), and the aim of this study was to elucidate the mechanisms underlying the anti-obesity effects of rose hip.

## Methods

### Experimental animal procedures

Eight-week old male C57BL/6 J mice were purchased from Taconic, Denmark. The animals were maintained in a temperature-controlled room (22 °C) with a 12:12 h light:dark cycle with free access to food and water. After one week of acclimatization, mice were randomly divided in two groups (*n* = 12) and fed a control HFD (CTR) or a HFD supplemented with rose hip (RH) for 3 months. The study was approved by the Local Animal Ethics Committee (Lund, Sweden).

### Diets

The two experimental diets contained 45 energy % from fat (Research diets, New Brunswick, NJ, USA) (Table 1). Rose hip powder was obtained from Orkla ASA, (Oslo, Norway) and analyzed by Eurofins Food & Agro Testing (Sweden). Based on its composition (Table 2), the macronutrient content was balanced in the two diets.

**Table 1:** Diet composition

Ingredients (g)	CTR	RH
Casein	200	191.3
L-Cysteine	3	3
Corn Starch	50	-
Maltodextrin 10	100	83
Glucose	21.6	-
Fructose	23.4	-
Sucrose	175	148.1
Cellulose	97.4	-
Soybean Oil	25	16.2
Lard	177.5	177.5
Mineral Mix	10	10
Dicalcium phosphate	13	13
Calcium Carbonate	5.5	5.5
Potassium Citrate	16.5	16.5
Vitamin Mix	10	10
Choline Bitartrate	2	2
Rose hip	-	295
Total (g)	929.9	971.1
Protein (Kcal %)	18	18
Carbohydrates (Kcal %)	37	37
Fat (Kcal %)	45	45
Kcal/g	4.4	4.2

**Table 2:** Rose hip powder analysis (per 100 g)

Moisture content	8.53 g ± 10%
Ash	5.76 g ± 10%
Fat	2.98 g ± 10%
Protein	2.62 g ± 10%
Carbohydrates (calculated)	47.1 g
Fiber	33.0 g ± 15%
Fructose	7.93 g ± 15%
Glucose	7.32 g ± 15%
Sucrose	9. 11 g ± 15%
Saturated fatty acids	27.7%
Monounsaturated fatty acids	9.7%
Polyunsaturated fatty acids	51.8%
Ascorbic acid	440 mg ± 10%

### Glucose tolerance test

An intraperitoneal glucose tolerance test (IPGTT) was performed on 6-hr-fasted mice by injecting 2 g/Kg of D-(+)-glucose (SIGMA-Aldrich, St. Louis, MO) followed by blood sampling from the saphenous vein in heparin-coated tubes at 0, 30, 60 and 120 min. Blood samples were centrifuged and plasma was collected and analyzed with Infinity™-Glucose (Fisher Diagnostic, Middletown – VA) according to the manufacturer’s instructions.

### Insulin tolerance test

An intraperitoneal insulin tolerance test (IPITT) was performed on 4-hr-fasted mice by injecting 0.75 U/kg of insulin (Actrapid®, Novo Nordisk, Denmark). Blood samples were collected at 0, 15, 30 and 60 min and blood glucose levels were determined with Accu-check Aviva (Roche Diagnostic Gmbh, Germany).

### Plasma analysis

At the end of the study blood was withdrawn by orbital puncture and collected in heparin-coated tubes from 10-hr fasted isofluorane-anesthetized mice. The blood was immediately centrifuged and plasma was collected, snap-frozen and stored at −80 °C. To assess plasma insulin, total cholesterol and triglycerides levels, Insulin ELISA assays (Mercodia, Sweden), Infinity™-Cholesterol and Infinity™-Triglycerides assays (Fisher Diagnostic, Middletown – VA) were used according to the manufacturer’s instructions.

### Indirect gas calorimetry and behavioral monitoring

Whole-body energy metabolism, food and water intake as well as voluntary locomotor and wheel activity were measured with PhenoMaster/LabMaster Home cage System (TSE-systems, Germany). Mice were weighed and acclimatized for two days prior to the experiments. All parameters were recorded every 15 min for 24 h. Two 24-h experiments were carried out and averaged for each mouse.

### Treadmill and indirect calorimetry measurements

The experiment was performed with a CaloTreadmill (TSE systems - Germany) set at a 20% inclination after a four-day training. EE, oxygen and carbon dioxide fractions were continuously monitored and the respiratory exchange ratio (RER) was calculated. Protocol run: after placing the mice inside the treadmill, the speed slightly increased from 0–14 m/min for 5 min, then from 14–18 m/min for 15 min followed by constant running at 18 m/min for 28 min. After that, the speed decreased from 18 to 0 m/min in 1 min. Exhaustion was defined as the inability to continue regular running.

### Oxygen consumption in WAT and BAT

The Clark oxygen sensor electrode (DW1, Hansatech Instruments, Norfolk, UK) was mounted in a chamber according to the manufacturer’s instructions and connected to a computer-operated control unit to register the cellular respiration (Oxygraph software, Hansatech). Prior to the experiment, the oxygen electrode was calibrated in Krebs Ringer HEPES (KRH) buffer (25 mM HEPES pH 7.5, 120 mM NaCl, 4.74 mM CaCl_2_, 2 mM glucose, 200 μM adenosine, 1% fatty acid free BSA) at 37 °C. A 2-point calibration was performed between the oxygen levels of air-saturated buffer and zero oxygen buffer. Perigonadal WAT and interscapular BAT were excised and immediately placed in KRH buffer. For this experiment a separate set of female mice were used. These mice were fed CTR and RH diets for 17 weeks. The diets were similar to the ones described above except for that they contained 41 energy % fat and that the ascorbic acid present in the RH was compensated for in the CTR diet. The tissues were analyzed for oxygen consumption within 2 h after excision. KRH buffer (500 μl/experiment) was pre-warmed at 37 °C in the oxygraph chamber and the measurement was started by establishing a stable background. A piece of WAT (62 ± 7 mg) or BAT (15 ± 4 mg) was minced 30 times with a pair of scissors and thereafter added to the KRH buffer in the chamber. The samples were continuously stirred with a magnetic stirrer and the lid of the chamber was adjusted to the sample volume. The oxygen consumption was measured during the first 6 min after addition of the tissue and expressed as nmol/min/mg tissue (after subtraction of background).

### Bomb calorimetry

The gross energy content in feces was determined with a bomb calorimeter (Parr Instrument Company - USA). Feces was collected from single-caged mice, freeze-dried for 24 h, and a pellet of approximately 1 g was made and burnt into the 1108P Oxygen Bomb (Parr Instrument Company - USA) following the manufacturer’s instruction.

### RNA preparation and real-time quantitative PCR

Total RNA was isolated from the tissue using Qiazol lysis reagent (Quiagen Sciences, USA). Two μg of RNA were treated with DNase I (DNase I amplification grade; Invitrogen, CA) and then reversely transcribed using random hexamers (Amersham Biosciences, NJ) and SuperScript II reverse transcriptase (Invitrogen, CA). Quantitative real-time polymerase chain reactions were performed using ABI PRISM 7900 System with TaqMan primers bmp7 (Mm00432102_m1), ppargc1a (Mm01208835_m1), fgf21 (Mm00840165_g1), elovl3 (Mm00468164_m1), rps29 (Mm02342448_gH), tbp (Mm01277042_m1), cidea (Mm00432554_m1), car4 (Mm00483021_m1), tfam (Mm00447485_m1), nrf1 (Mm01135606_m1) and reagents (Applied Biosystems, CA); Qiagen SYBR green primers were Prdm16 (QT00148127), tbx15 (QT00148127), zic1 (QT00173502), tcf21 (QT00100688), ucp1 (QT00097300), cpt1 (QT00172564) and tbp (QT00198443). Each reaction was performed in duplicate and results were normalized to the geometric average of two internal controls (Rps29 and Tbp).

### Western blotting

Approximately 50 mg of tissue were homogenized in lysis buffer (50 mM Tris–HCl, pH 7.5, 1 mM EGTA, 1 mM EDTA, 1% NP40, 1 mM Na-orthovanadate, 40 mM NaF, 4 mM Na-pyrophosphate, 0.27 M sucrose, 1 mM DTT, 20 μg/ml leupeptin, 10 μg/ml antipain and 1 μg/ml pepstatin) and subsequently centrifuged at 12,000 g for 20 min at 4 °C. Protein concentration of the supernatant was determined using BCA-assay (Pierce) and 20 μg of proteins were resolved on NuPAGE 4–12% Bis-Tris Gel (Life technologies – Carlsbad, CA) and electroblotted to nitrocellulose membranes (Amersham, GE Healthcare – UK). Primary antibodies used were: Cidea (Abin1858406 – Antibodies online), beta-actin (A5441 – Sigma), pAMPK (CST2535 – Cell Signaling), AMPK (CST2603 – Cell Signaling), PGC1α (Ab54481 – Abcam). Secondary antibodies were: sheep anti-mouse (NA931V – GE Healthcare) and goat anti-rabbit (31460 – Thermo Fisher).

### Statistical analysis

Results are presented as means ± SD. Statistical analysis was performed with Graph-Pad Prism 6 using unpaired Student’s *t*-test and two-way ANOVA followed by *Tukey's post*-*hoc test* for the treadmill data set. Values of *p* < 0.05 were considered statistically significant.

## Results

### RH supplementation prevents weight gain and exerts anti-diabetic effects

**Fig. 1:**
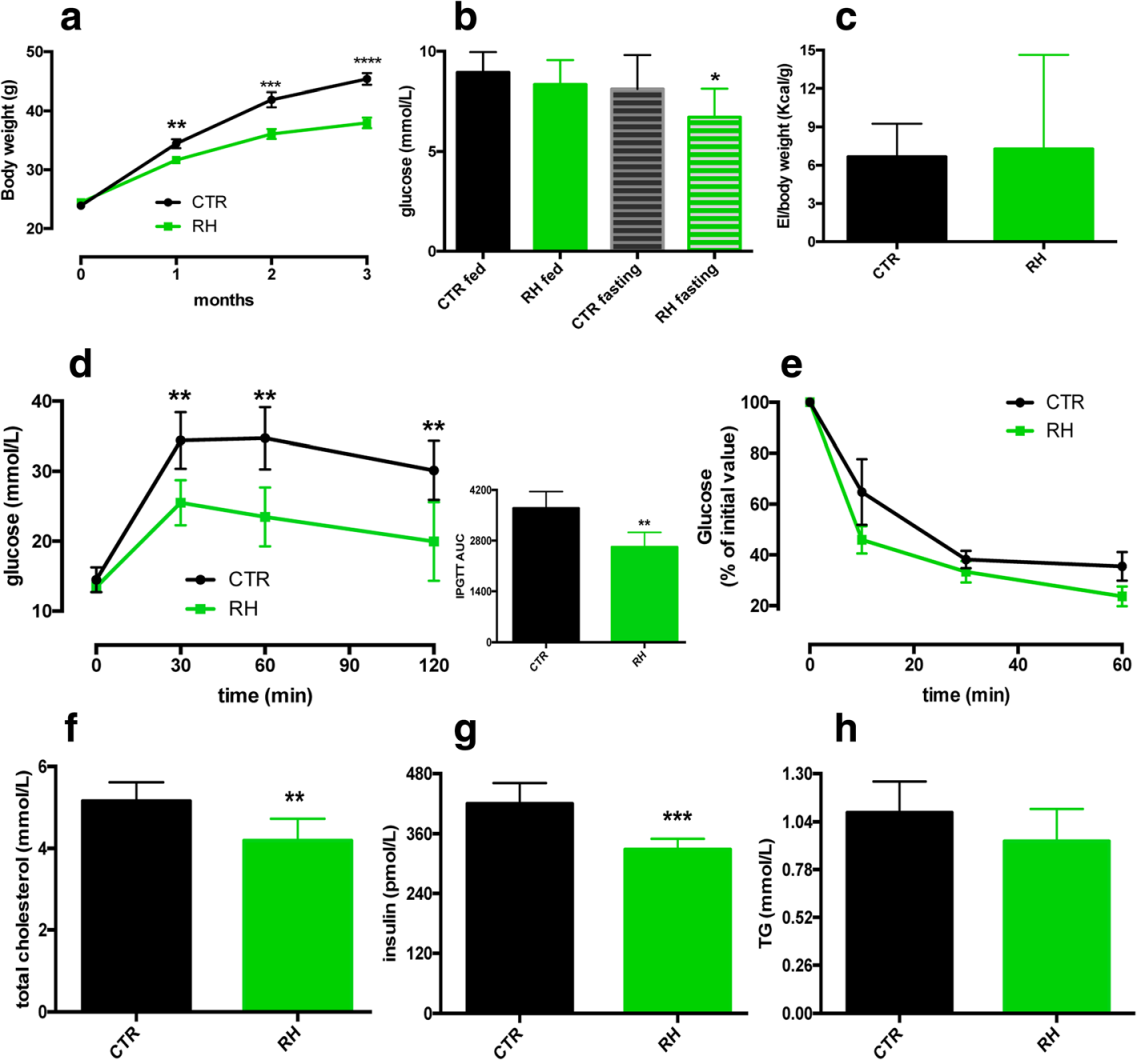
Dietary RH intake prevents diet-induced obesity and reduces glucose and insulin levels. **a** RH feeding prevented body weight gain and **b**) reduced blood glucose levels (*n* = 16) during high-fat feeding. **c** No differences were found in energy intake between the two groups of mice (*n* = 10). **d** Dietary RH improved glucose disposal after an IPGTT (*n* = 6) but **e**) had no effect upon an IPITT (*n* = 6). **f**-**h** RH effects on plasma cholesterol, insulin and TG levels (*n* = 6). *P < 0.05; **p < 0.005; ***p < 0.001 and ****p < 0.0001 vs CTR

At the end of the study, mice fed RH showed reduced body weight (Fig. 1a) and fasting glucose levels (Fig. 1b), despite similar EI between the two groups (Fig. 1c). An IPGTT revealed that RH-fed mice have increased glucose disposal (Fig. 1d) while no differences were found after an IPITT (Fig. 1e). Compared to the CTR group, RH supplementation markedly reduced plasma total-cholesterol and insulin levels (Fig. 1f-g). Triglycerides levels were comparable between the two groups (Fig 1h).

### RH feeding increases EE, VO_2_ and VCO_2_

**Fig. 2:**
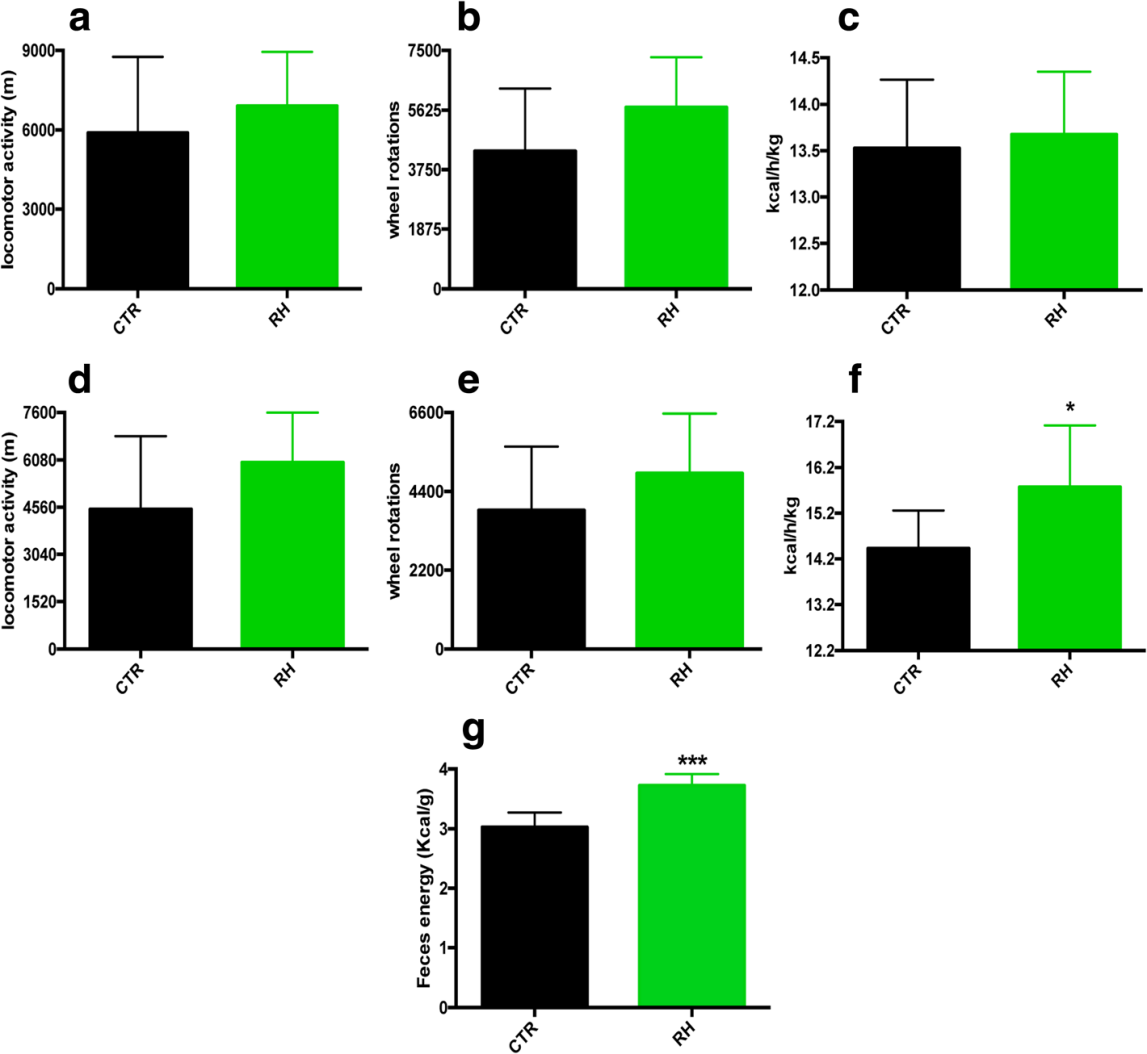
RH intake increases EE during the dark cycle. **a**-**c** Locomotor activity, wheel rotations and EE during 24-h measurements, and **d**-**f**) during the 12-h dark phase only (*n* = 8–9). **g** Increased fecal energy content upon RH feeding (*n* = 6). *P < 0.05 and *** *p* < 0.001 vs CTR

**Fig. 3:**
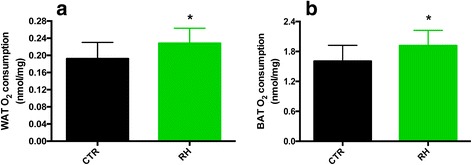
Increased oxygen consumption in WAT and BAT obtained from RH-fed mice. **a** WAT and **b**) BAT O_2_ consumption (*n* = 9–10) was increased upon RH feeding. *P < 0.05 vs CTR

24-h calorimetric cages experiments showed no differences in locomotor activity, wheel running or EE (Fig. 2a-c) between the two groups of mice. However, when considering the dark phase only, RH-fed animals displayed again similar locomotor (Fig. 2d) and wheel activity (Fig. 2e), but showed markedly higher EE (Fig. 2f), suggesting that the increased EE was not attributed to hyperactivity. Furthermore, analysis of the feces revealed a significantly higher energy content following RH feeding (Fig. 2g). Additionally, a previous oxygen consumption experiment performed on ex vivo WAT and BAT from female mice indicated that mitochondrial respiration was increased upon RH-feeding (Fig. 3a and b), corroborating the new in vivo results.

### RH supplementation increases metabolic rate during treadmill exercise

**Fig. 4:**
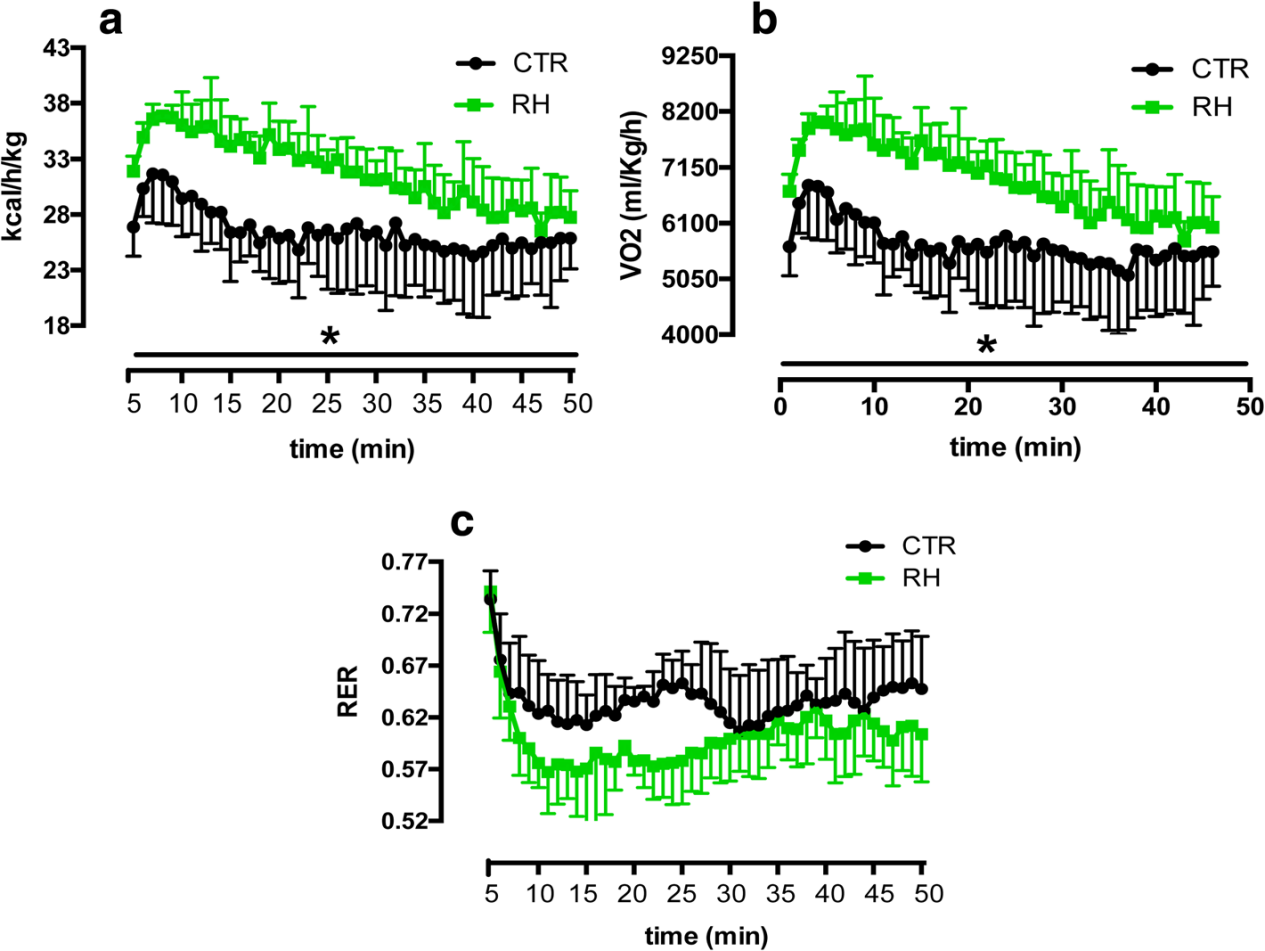
RH supplementation increases metabolic rate during treadmill exercise. RH feeding led to a significantly increased EE (**a**) and VO_2_ (**b**), while RER (**c**) was comparable between the two groups during treadmill running (*n* = 5). *P < 0.05 vs CTR

Since physical exercise increases EE [35, 36] and activates the browning program [18], we challenged the two groups of mice with treadmill running. Again, indirect calorimetry showed that RH-fed mice had considerably higher EE (Fig. 4a) and VO_2_ (Fig. 4b), while no differences were found in the RER (4c). Interestingly, one mouse of the RH-group was not able to conclude the run, while another one completed the test but reached the bottom of the treadmill a few times before immediately restarting the running. All the CTR-mice concluded the test without any problem.

### RH feeding upregulates *ucp1* and other BAT and brite markers in WAT

**Fig. 5:**
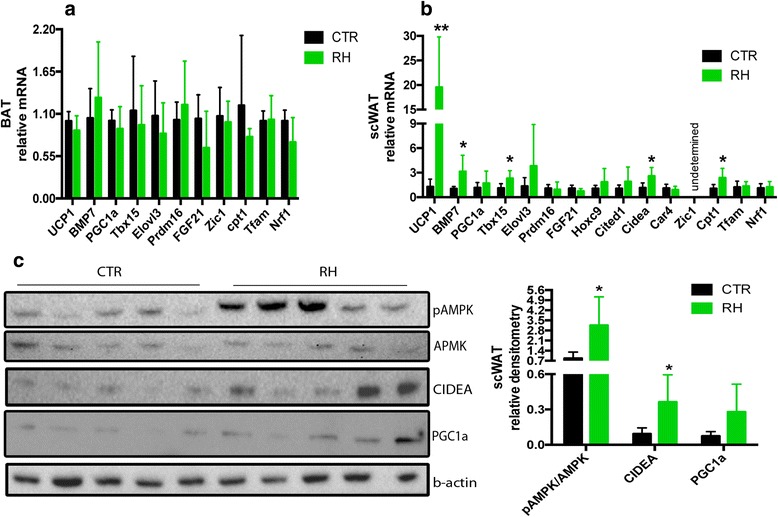
RH feeding upregulates BAT markers in scWAT. **a** Dietary RH had no effect on BAT- and brite-gene expression in the interscapular adipose tissue, but **b**) increased the expression of some of these markers in the scWAT. **c**) Western blot analysis of the scWAT revealed increased AMPK phosphorylation and higher CIDEA levels compared to the CTR mice. *N* = 5 for all, **p < 0.005 and *p < 0.05 vs CTR

In order to investigate if dietary RH is able to stimulate BAT thermogenic activity, we analyzed the expression of selected genes of the interscapular brown adipose depot. Compared to the CTR group, no differences were found in the expression of *ucp1* or any of the other genes analyzed (Fig. 5a). Next, we examined the expression of BAT- and brite-markers in the subcutaneous inguinal WAT (scWAT). In this fat depot, *ucp1* was markedly upregulated by RH, as well as *bmp7*, *tbx15* and *cidea* (Fig. 5b). Furthermore, among markers of mitochondrial oxidation, *cpt1* was significantly increased (Fig. 5b). The expression of *zic1*, a typical BAT marker, was undetected in both groups (Fig. 5b). Western blotting analysis revealed that dietary RH markedly upregulated CIDEA and increased the phosphorylation of AMPK (Fig. 5c), while the tendency for increased PGC1α levels did not reach significant differences (Fig. 5c).

## Discussion

Obesity develops from an unbalance of energy homeostasis when EI exceeds EE. It represents a major risk factor for the development of type 2 diabetes, dyslipidemia, cardiovascular disease and several other conditions. Our previous studies indicate that RH is able to prevent weight gain [26], so we aimed at investigating the mechanisms through which RH exerts these effects by challenging C57BL/6 J mice with a HFD supplemented with RH.

Compared to the CTR group, RH supplementation prevented body weight gain and reduced glucose, insulin and cholesterol levels even though the two groups showed similar EI. We then used calorimetric cages to measure in vivo indirect-gas calorimetry. Despite no differences were found during the 24-h measurement, RH-fed mice displayed higher EE during the dark phase, while spontaneous wheel and locomotor activities were comparable between the two groups. Bomb calorimetry analysis showed that feces of RH-fed mice had higher energy content compared to the CTR group, indicating that RH decreases intestinal energy absorption. This effect most likely contributes to the anti-obesity effect of RH. In addition, mice fed RH exhibited higher metabolic rate also during intense physical exercise. The fact that one mouse of the RH-group ended the treadmill test before the end of the experiment, and another one was close to, but did not reach exhaustion, could reflect an insufficient delivery of adipose tissue-derived fatty acids to skeletal muscle, as a result of higher mitochondrial uncoupling capacity due to browning.

Quantitative PCR analysis revealed no gene expression differences in the interscapular BAT between the two groups of animals, but showed that dietary RH strikingly upregulates *ucp1* and other genes such as *bmp7*, *tbx15, cidea* and *cpt1* in the scWAT, indicating attainment of brown-like characteristics of the subcutaneous adipocytes. BMP7 is a bone morphogenic protein known for promoting brown adipogenesis and increasing the expression of UCP1; it also induces mitochondrial biogenesis through p38 mitogen-activated protein (MAP) kinase and PGC1-dependent pathways [37]. As a matter of fact, *bmp7*-null mice show complete absence of UCP1, while overexpression of BMP7 increased brown fat mass and EE and reduced weight gain [37, 38]. Included among the genes upregulated by RH feeding is *tbx15,* a transcription factor that is required for adipogenesis in BAT and inguinal WAT, but not in epididymal WAT [39]. It has been reported that *tbx15* knockdown reduced the expression of PGC1α and UCP1, but had no effects on white epididymal adipocytes, suggesting that TBX15 may be essential for the development of adipogenic and thermogenic programs in fat depots capable of developing brown adipocyte features [40]. CIDEA is a multifunctional protein involved in apoptosis and transcriptional regulation. Its cold-induced increment in WAT suggests that CIDEA could be an important effector during the browning process. Indeed, in *ucp1* knockout mice, *cidea* is dramatically induced in WAT by cold exposure, indicating that it participates in cold acclimatization mechanisms independent of UCP1 [41]. However, gene expression of other “brite” markers such as *hoxc9*, *fgf21, cited1* and *car4* did not change upon RH feeding.

Together with CIDEA protein levels, phosphorylation of AMPK was also elevated in the RH group. Much of the interest in AMPK generated in the past years is due to the observation that its activity is reduced in most genetic models of obesity [42]. Very recently, Wang and coworkers demonstrated that resveratrol induces brown-like adipocyte formation in white fat through activation of AMPK [19]. AMPK can also phosphorylate PGC1α to increase mitochondrial biogenesis [43], and another study showed that AMPK is essential for brown adipocyte differentiation and browning of WAT [44]. Thus, the increased AMPK phosphorylation observed upon RH feeding may be an important mechanism for reducing obesity and increasing EE, but it requires further investigation.

## Conclusions

In the present study we identify two possible mechanisms whereby RH may exert anti-obesity effects. First, dietary intake of RH induces browning of scWAT through upregulation of some BAT markers and increased phosphorylation of AMPK. Second, dietary intake of RH appears to decrease intestinal energy absorption. Thus, RH intake exerts anti-obesity through effects on both EE and EI. Confirmatory experiments in human subjects as well as complementary mechanistic studies are required in order to grant RH a “brite” future as a dietary supplement to prevent and treat obesity and related metabolic disorders.

## Abbreviations

BAT: brown adipose tissue
EE: energy expenditure
EI: Energy intake
IPGTT: intraperitoneal glucose tolerance test
IPITT: intraperitoneal insulin tolerance test
RER: respiratory exchange ratio
RH: rose hip
sc WAT: subcutaneous white adipose tissue
WAT: white adipose tissue
